# In Response

**DOI:** 10.4269/ajtmh.16-0985b

**Published:** 2017-03-08

**Authors:** Kittiyod Poovorawan

**Affiliations:** 1 Department of Clinical Tropical Medicine, Faculty of Tropical Medicine, Mahidol University, Bangkok, Thailand. E-mail: kittiyod.poo@mahidol.ac.th

Dear Sir:

The letter to the editor entitled “Acute liver failure and dengue: alcohol matters” by Debes and Ashhab^1^ has addressed the important issue of alcohol intake on potentially more severe liver involvement in dengue infection.

Data from Thailand’s nationwide database have revealed that alcoholic liver disease is the major identified cause of cirrhosis in Thailand.^2^ Alcohol consumption also plays an indirect concomitant role in contracting dengue infection due to the increased risk of mosquito bite and resultant exposure to dengue virus (DENV) and mosquito-borne disease.^3^ However, previous studies determining the direct effects of alcohol consumption on the severity of liver involvement in dengue infection are lacking. Even without the alcohol factor, dengue infection is a cause of acute liver failure, based on the study of acute liver failure caused by dengue infection among Thai children.^4^,^5^

The retrospective design of the recent study by Kye Mon and others is a limitation. Evidence of prior alcohol consumption was based on medical records, which frequently do not quantify the amount of alcohol consumed, the time during which it was consumed, and the overall duration and level of alcohol consumption, to determine alcohol intake. Therefore, this parameter was not included in the present study. The effects of alcohol consumption on the severity of liver involvement in dengue infection require further investigation.

A recent study has demonstrated the utility of human pluripotent stem cell-derived hepatocyte-like cells as an in vitro model for DENV infection,^6^ and may be suitable for evaluating the direct effects of alcohol in vitro. A future prospective study to evaluate the effects of alcohol consumption in dengue and various other tropical infections should reduce the current knowledge gap and may lead to new mechanisms for prevention.
